# Exosomes from miRNA‐126‐modified endothelial progenitor cells alleviate brain injury and promote functional recovery after stroke

**DOI:** 10.1111/cns.13455

**Published:** 2020-10-03

**Authors:** Jinju Wang, Shuzhen Chen, Wenfeng Zhang, Yanfang Chen, Ji C. Bihl

**Affiliations:** ^1^ Department of Pharmacology & Toxicology Boonshoft School of Medicine Wright State University Dayton OH USA; ^2^ Department of Biomedical Science Joan C. Edwards School of Medicine Marshall University Huntington WV USA

**Keywords:** diabetes, endothelial progenitor cells, exosomes, ischemic stroke, miRNAs

## Abstract

**Aims:**

We previously showed that the protective effects of endothelial progenitor cells (EPCs)‐released exosomes (EPC‐EXs) on endothelium in diabetes. However, whether EPC‐EXs are protective in diabetic ischemic stroke is unknown. Here, we investigated the effects of EPC‐EXs on diabetic stroke mice and tested whether miR‐126 enriched EPC‐EXs (EPC‐EXs^miR126^) have enhanced efficacy.

**Methods:**

The db/db mice subjected to ischemic stroke were intravenously administrated with EPC‐EXs 2 hours after ischemic stroke. The infarct volume, cerebral microvascular density (MVD), cerebral blood flow (CBF), neurological function, angiogenesis and neurogenesis, and levels of cleaved caspase‐3, miR‐126, and VEGFR2 were measured on day 2 and 14.

**Results:**

We found that (a) injected EPC‐EXs merged with brain endothelial cells, neurons, astrocytes, and microglia in the peri‐infarct area; (b) EPC‐EXs^miR126^ were more effective than EPC‐EXs in decreasing infarct size and increasing CBF and MVD, and in promoting angiogenesis and neurogenesis as well as neurological functional recovery; (c) These effects were accompanied with downregulated cleaved caspase‐3 on day 2 and vascular endothelial growth factor receptor 2 (VEGFR2) upregulation till day 14.

**Conclusion:**

Our results indicate that enrichment of miR126 enhanced the therapeutic efficacy of EPC‐EXs on diabetic ischemic stroke by attenuating acute injury and promoting neurological function recovery.

## Graphical abstract

Schematic shows the mechanism of EPC‐EXs^miR‐126^ on treating ischemic stroke in diabetes. Overexpression of miR‐126 enhanced the therapeutic effects of EPC‐EXs on diabetic ischemic stroke by reducing cell apoptosis in acute phase and promoting angiogenesis and neurogenesis to promote neurological function recovery in chronic phase.

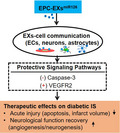

## 1 INTRODUCTION

Ischemic stroke is a loss of neurologic function due to an occluding of blood vessels in or leading to the brain; ischemic stroke remains one of the most severe worldwide health problems. Diabetes mellitus leads to 3‐4 times higher risk of IS than nondiabetes Mellitus, since the combinations of impaired endothelial dysfunction and decreased angiogenesis exaggerate the cerebral damage. Surgical treatments like tirofiban infusion have risks associated with elevating hemorrhagic, and angioplasty with a high chance of thrombosis.^1^ Currently, effective neuroprotective drugs are unavailable. Thus, theoretically, a therapeutic strategy targeting both acute vascular protection and later neurological recovery phases might be highly significant.

Stem cell‐based therapy is one promising avenue for ischemic stroke due to multiple benefits, including protective effects on the vascular and neuronal unit through their released growth factor and angiogenic factor.^2^ Several types of stem/progenitor cells such as mesenchymal stem cells and endothelial progenitor cells (EPCs) have been investigated to determine the feasibility and efficacy of therapeutic function in stroke.^3^, ^4^ Among them, EPCs hold great potential because of their abilities for vascular and neuronal protection, repair, and regenesis.^5^ Our previous studies that have demonstrated that infusion of EPCs provides therapeutic effects on ischemic stroke by cerebrovascular protection in the acute phase and promoting neurological recovery in chronic phases.^6^, ^7^

Exosomes (EXs), a type of extracellular vesicles, have been shown as an effective way of cell‐cell and organ‐organ communication by delivering their cargoes, such as proteins, mRNAs, and microRNAs (miRs).^8^, ^9^ Recently, increasing data suggest that the benefits of stem cell‐based therapy are due to their released extracellular vesicles, including EXs.^10^, ^11^ Mesenchymal stem cells released EXs have been shown to promote neurovascular plasticity and regulate axon outgrowth after stroke in rats via transfer of miR133b to neural cells.^12^, ^13^ The mesenchymal stem cells released EXs are also shown to be able to improve diabetes‐induced cognitive impairment of STZ‐diabetic mice.^14^ EPC‐released microvesicles have been demonstrated to reduce ischemia/reperfusion‐induced injury in hindlimb and kidney by transferring their carried miR‐126.^15^, ^16^ The umbilical cord blood‐derived EPC‐EXs are able to stimulate the angiogenic activity of endothelial cells and thereby facilitating wound healing and regeneration in STZ‐induced diabetic rats.^17^ Our group found that EPC‐released microvesicles protected the endothelial cell against hypoxia/reoxygenation (H/R)‐induced dysfunction and injury.^18^ More recently, we further demonstrated that EPC‐released microvesicles enhance the viability and function of EPCs in diabetes. The underlying mechanism is related to their carried miR‐126 which further activates the vascular endothelial growth factor receptor 2 (VEGFR2) signal transduction.^19^ However, it remains unknown whether (a) intravenous injection of EPC‐EXs has therapeutic effects on diabetic ischemic stroke and (b) enrichment of miR‐126 in EPC‐EXs could enhance the effect.

In the present study, we determined the therapeutic effects of EPC‐EXs on diabetic ischemic stroke and explored whether the miR‐126/VEGFR2 pathway is responsible for the beneficial effects.

## 2 MATERIALS AND METHODS

### 2.1 Experimental animals

A total of 143 db/db type II diabetic mice (8‐10 weeks, weight 42‐48 g) bought from Envigo were subjected to MCAO surgery for inducing an ischemic stroke. Elven mice died of surgery intervention, and the remaining 132 were used for the experiments. For donating EPCs, a total of 62 same gene background mice (db/c, 8‐10 weeks, weight 25‐32 g) from the Jackson Laboratory (Bar Harbor, ME) were used. To measure the angiogenesis and neurogenesis, BrdU (IP, 65 μg/g per day) was injected into mice for 7 continuous days.^6^, ^7^ All protocols were approved by the Wright State University Laboratory Animal Care and Use Committee, and all experiments were conducted in accordance with Guide for the Care and Use of Laboratory Animals (published by the National Research Council) and reported in compliance with ARRIVE guidelines.

### 2.2 EPC culture, characterization, and transfection

EPCs were cultured from the bone marrow of C57BL/6 mice as we previously reported.^6^, ^7^, ^20^ Cultured EPCs were characterized by Dil‐acLDL and Bs‐Lectin staining as previously reported.^6^, ^20^ To upregulate the levels of miR‐126 in EPC‐EXs, EPCs were transfected with miR‐126 scramblers or mimics (1 nmol/L, Applied Biosystems) using DharmaFECT1 transfection reagent (Dharmacon) for 24 hours to generate miR‐126 modified EPCs.^19^ The EXs from miR‐126 scrambler and miR‐126 mimic treatment groups were defined as EPC‐EXs and EPC‐EXs^miR126^, respectively.

### 2.3 EPC‐EX isolation and labeling

EPC‐EXs were collected from the cell culture medium of EPCs per our previous publication.^21^ One portion of the pelleted EPC‐EXs was resuspended with phosphate‐buffered saline (PBS) for size and concentration analyses by using nanoparticle tracking analysis system (NTA). The other portion of the EPC‐EXs were labeled with PKH26 (2 × 10^‐6^ mol/L, Sigma‐Aldrich, St. Louis, MO)^22^ and resuspended with PBS for infusion.

### 2.4 Concentration and size analyses of EPC‐EXs

The concentration and the size range of the EPC‐EXs were measured by NanoSight NS300 (Malvern Instruments, Malvern, UK). For analyzing the level of EPC‐EXs from the brain tissue, we applied MACS combining with fluorescence NTA by using EPC specific markers (CD34 and KDR) as we previously reported.^21^

### 2.5 Middle cerebral artery occlusion (MCAO) surgery and EPC‐EX infusion

Db/db diabetic mice were induced an ischemic stroke by MCAO surgery as our previous reports.^20^, ^23^ Pain and discomfort were minimized by using Buprenorphine (0.1 mg/kg, sc) and Carprofen (5 mg/kg, sc). Two hours after MCAO, mice (n = 11/group) were randomly injected with vehicle (PBS), EPC‐EXs, or EPC‐EXs^miR126m^ (1 × 10^11^ EXs/100 µL, comparable to 50 μg total exosome protein, per mouse) via the tail vein. The dose was chosen based on previous publications on EX infusion for treating stroke or brain injury^12^, ^24^ as well as our pilot study on dosage. Mice were euthanized by injection of ketamine: xylazine mixture (100:8 mg/kg, IP) at day 2 and day 14 after EPC‐EX infusion. The expression of miR‐126 in the brain was determined by a qRT‐PCR method.^19^, ^21^

### 2.6 Isolation of EXs from brain tissues

The EXs were isolated and purified from brain tissues according to a previously published protocol with slight modification.^25^ In brief, brains were dissected and gently rinsed in PBS, and incubated in 7 mL of papain (20 units/mL, Worthington) in Hibernate‐A for 20 minutes at 37°C. Hibernate‐A containing complete protease inhibitor mixture (14 mL, Roche) was used to stop the reaction. The tissue was gently disrupted by pipetting, followed by centrifugations at 300 *g* for 30 minutes at 4°C to remove pellets containing cells, and 2000 *g* for 20 minutes 4°C to discard cell debris, and 20 000 *g* for 70 minutes at 4°C to discard microvesicles. The supernatant was centrifuged at 100 000 *g* for 90 minutes at 4°C to pellet EXs after passing through a 0.22‐µm syringe filter (Millex‐GP, Millipore). Pelleted EXs were suspended with 100 µL PBS (filtered through 20 nm filter) for NTA analysis.

### 2.7 Measurement of cerebral blood flow (CBF), infarct volume, and cerebral microvascular density (MVD)

The CBF of mice was determined by the PeriCam PSI System (Perimed, Sweden) and calculated as reported previously.^26^ The infarct volume and MVD were evaluated by cresyl violet (CV) staining^27^ and CD31 staining.^6^, ^7^, ^20^, ^23^ The area of infarction was calculated by subtracting the area of the nonlesioned ipsilateral hemisphere from the total area of the contralateral hemisphere. The microvascular was counted when its length is twice its width by using Image J software (NIH). The mean density of MVD from six sequential brain sections of the individual mouse was calculated and expressed as numbers/mm^2^.

### 2.8 Functional evaluation of neurological deficits and function

The neurological deficit scores (NDS) were evaluated on days 2 and 14 after EPC‐EX infusion by using the 5‐point scale method as we previously described.^7^, ^23^ The sensorimotor deficits were assessed by the adhesive removal test and corner test as previously reported.^28^, ^29^ For the adhesive removal test, two different times were recorded, the time‐to‐contact and the time‐to‐remove the tape. First of all, a piece of adhesive tape was placed on each (right and left) forepaw. Mice were trained for 3‐5 days before stroke induction until the mice could take the adhesive tape off their paws within 12 seconds. For the corner test, two connected cardboard walls (30 × 20 × 1 cm) were taped together to form a 30° angle. To start the trial, mice were placed halfway into the apparatus facing the corner. The number of left turns out of 10 turn trials was recorded. All tests were performed by an investigator who was unaware of the grouping information.

### 2.9 Immunofluorescence analyses

Co‐localization of the injected EPC‐EXs with brain endothelial cells (CD31), neurons (NeuN), astrocytes (GFAP), and microglia (IBA‐1) on day 2 after stroke, as well as angiogenesis and neurogenesis in the peri‐infarct area on day 14 after stroke were determined as before.^6^, ^7^ In situ apoptosis was measured by terminal deoxynucleotidyl transferase (TdT) dUTP Nick‐End Labeling (TUNEL) assay kit (Roche, Switzerland) according to the manufacturer's instructions. In brief, brain slices (20 µm) mounted on gelatin‐coated slides and permeabilized with 0.1% TritonX‐100/0.1% sodium citrate for 2 minutes. And then the slides were washed and incubated with freshly prepared TUNEL reaction mixture in an incubator for 60 minutes at 37°C in the dark. Cell nuclei were statin with 4’, 6‐diamidino‐2 ‐phenylindole (DAPI, 1 µg/mL; Wako Pure Chemical Industries Ltd). Tissue samples were examined under a fluorescence microscope (EVOS, NY).

### 2.10 Western blotting

Proteins from the brain were isolated with lysis buffer (Roche Diagnostic) containing protease inhibitors. Antibodies against cleaved caspase‐3 (1:200; EMD Millipore), VEGFR2 (1:1000; Cell Signaling Technology), and β‐actin (1:4000; Sigma) were used.

### 2.11 Statistical analysis

The data of neurological deficit scores were expressed as median (range). All other data are presented as mean ± SD. Multiple comparisons between or among groups were analyzed by the Kruskal‐Wallis test followed by the Mann‐Whitney *U* tests. or 2‐way ANOVA followed by a Tukey post hoc test (SPSS 25, Chicago, IL, USA). The NDS was analyzed using the Kruskal‐Wallis test followed by Mann‐Whitney *U*‐test. Multiple comparisons between or among groups of the parametric data including infarct volumes, cell apoptosis, and effects of EPC‐EX treatment were assessed using one‐ or two‐way ANOVA, followed by Tukey test. For all tests, a *P* < 0.05 was considered significant.

## 3 RESULTS

### 3.1 Transfection of miR‐126 mimics increases the levels of miR‐126 in EPCs and EPC‐EXs

**Figure 1:**
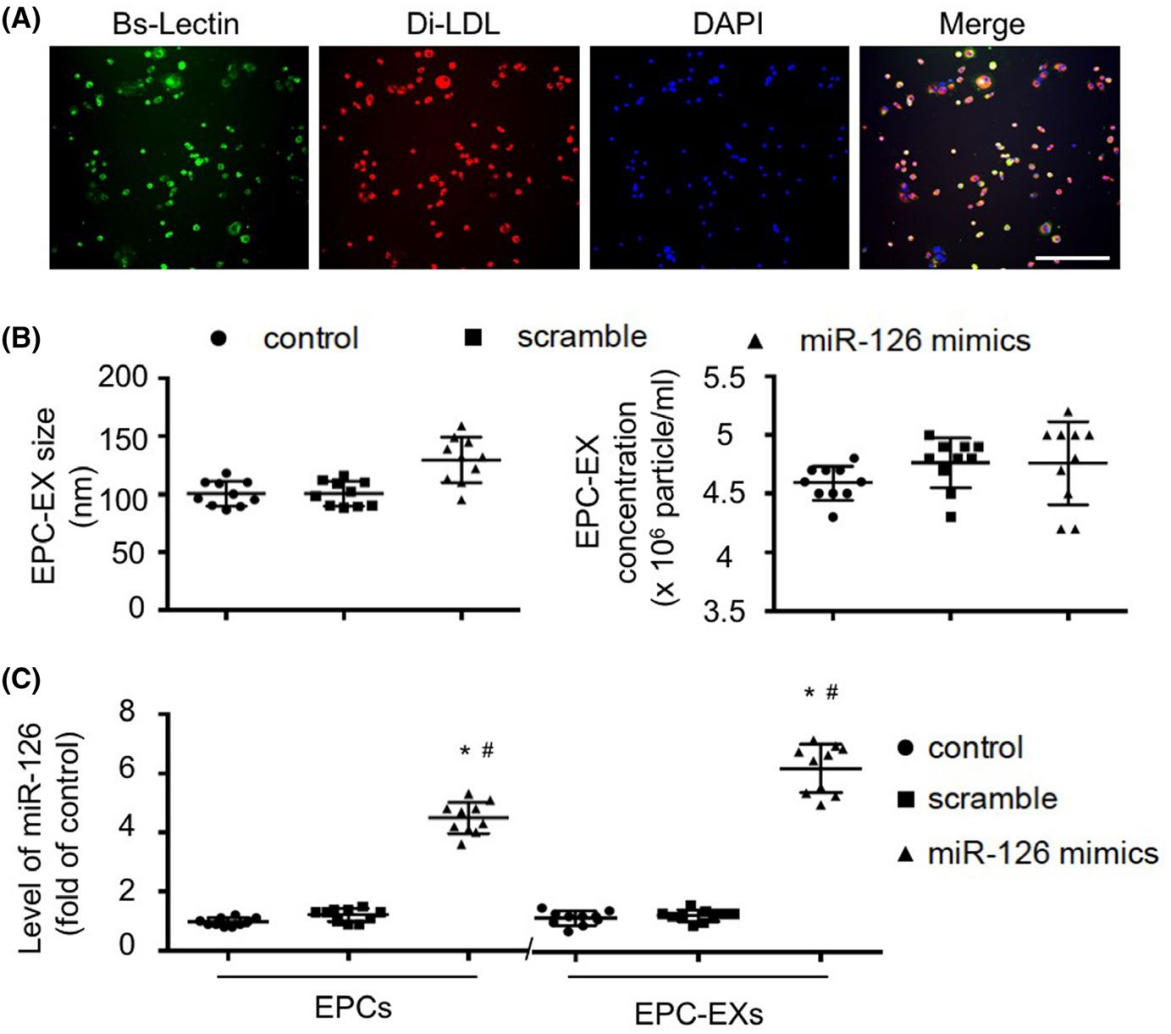
Characterization of EPCs and EPC‐EXs and generation of miR‐126 enriched EPC‐EXs. A*:* Characterization of EPCs by Bs‐Lectin labeling and Dil‐acLDL uptaking. Green: Bs‐Lectin; Red: Dil‐acLDL; Blue: DAPI for cell nucleus. Scale bars: 150 µm. B: NTA analysis of the concentration and size of EPC‐EXs. *C*: qRT‐PCR analysis of the level of miR‐126 in EPCs and EPC‐EXs. *P < 0.05 vs control or scrambler; ^#^ *P* < 0.05 vs EPCs; n = 10/group. Data are displayed as mean ± SD

As we reported previously,^6^, ^20^ BM‐derived EPCs were defined as cells uptaking Dil‐ac‐LDL and binding with Bs‐Lectin (Figure 1A). The EPC‐EXs were characterized by NTA showing that there were no differences in the size (around 100 nm) and concentration among the three types of EPC‐EXs (*P* > 0.05, Figure 1B). As shown in Figure 1C, miR‐126 mimics transfection significantly increased the level of miR‐126 by about 4‐fold in EPCs and 6‐fold in EPC‐EXs (*P* < 0.05). These data suggest that the miR‐126 can be enriched in EXs.

### 3.2 Infusion of EPC‐EXs^miR126^ is more effective than EPC‐EXs in decreasing ischemic injury in diabetic stroke mice

**Figure 2:**
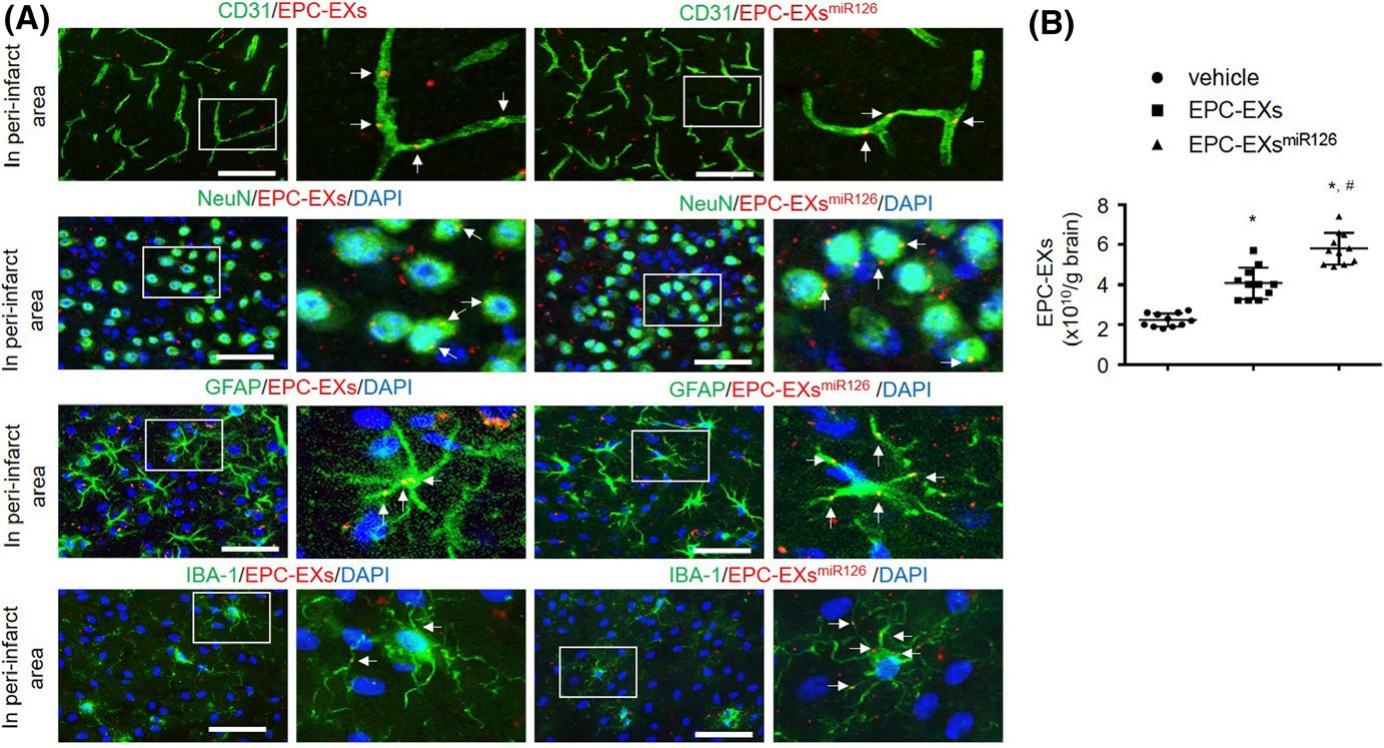
Intravenously injected EPC‐EXs merge with brain cells in the peri‐infarct area, and miR‐126 enrichment increased the recruitment of EPC‐EXs in the brain after ischemic stroke. A: PKH26 labeled EPC‐EXs (red) were injected via tail vein 2 h after MCAO‐induced ischemic stroke. The merging of EPX‐EXs with ECs (CD31, green), neurons (NeuN, green), astrocytes (GFAP, green), and microglia (IBA‐1, green) was determined on day 2 after EPC‐EX infusion. Right images are the enlarged view of the squared areas. Arrows (yellow) indicate the merging of EXs with cells, Bars: 80 μm for CD31; 40 μm for NeuN, GFAP, and IBA‐1. B*:* NTA quantification data showing the level of EPC‐EXs in the brain on day 2 after EPC‐EX infusion. *P < 0.05 vs vehicle; ^#^ *P* < 0.05 vs EPC‐EXs; n = 11/group. Data are displayed as mean ± SD

**Figure 3:**
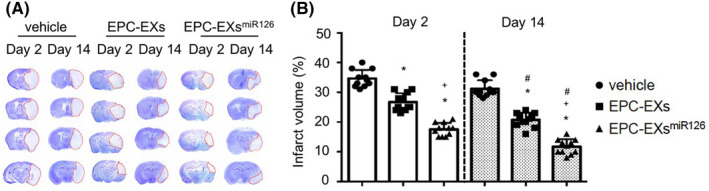
The effects of EPC‐EXs^miR126^ on infarct volume following ischemic stroke in diabetic mice. A: Representative images of CV staining for infarct volume. The stained area (blue) is the noninfarct area; while the unstained area (white) is the infarct area. B: Summarized data showing the infarct volume in different treatment groups following MCAO‐induced ischemic stroke. The infarct volume was presented as the % of the infarct area to the total area of the ipsilateral hemisphere. *P < 0.05 vs vehicle; ^#^ *P* < 0.05 vs Day 2; ^+^ *P* < 0.05 vs EPC‐EXs; n = 11/group. Data are displayed as mean ± SD

Representative images in Figure 2A shows that injected EPC‐EXs and EPC‐EXs^miR126^ merge with brain ECs, neurons, astrocytes, and microglia, dominantly in the peri‐infarct area. EPC‐EX infusion significantly increased the level of EPC‐EXs in the brain on day 2, while EPC‐EXs^miR126^ infusion further increased the EPC‐EX level (*P* < 0.05, Figure 2B). More interesting, our data showed that EPC‐EX infusion decreased infarct volume on both day 2 and day 14 (vs vehicle; *P* < 0.05). What's more, infusion of EPC‐EXs^miR126^ was able to further decrease the infarct size on day 2 (*P* < 0.05) and day 14 (vs EPC‐EXs; *P* < 0.05) (Figure 3).

### 3.3 Infusion of EPC‐EXs^miR126^ is more effective than EPC‐EXs in preserving CBF and MVD in diabetic stroke mice

**Figure 4:**
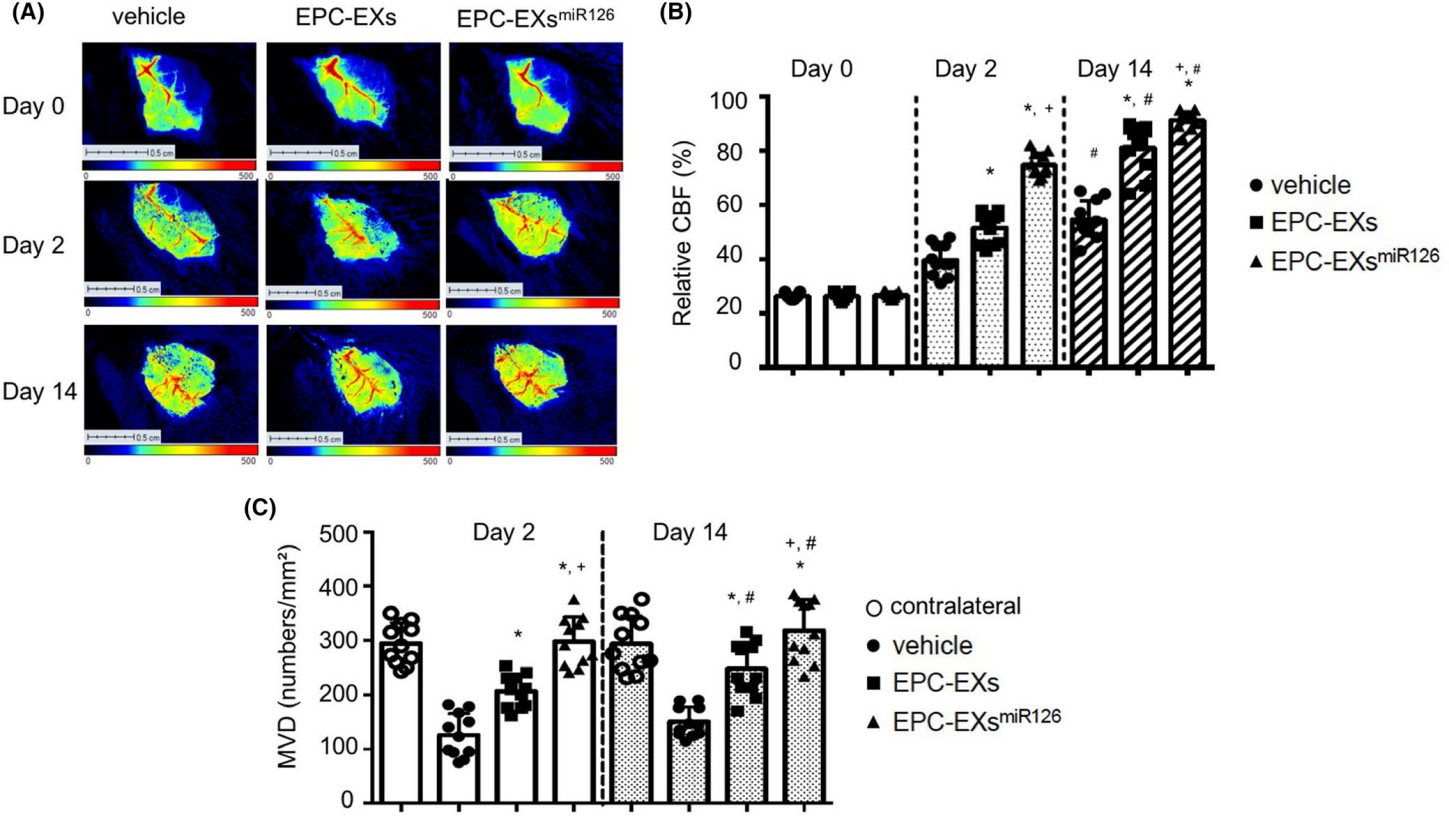
The effects of EPC‐EXs^miR126^ on CBF and MVD following ischemic stroke in diabetic mice. A: Representative images of CBF. B: Summarized data on CBF in different treatment groups following MCAO‐induced ischemic stroke. C: Summarized data on MVD in different treatment groups following MCAO‐induced ischemic stroke. *P < 0.05 vs vehicle; ^#^ *P* < 0.05 vs Day 2; ^+^ *P* < 0.05 vs EPC‐EXs; n = 11/group. Data are displayed as mean ± SD

As shown in Figure 4A,B, the data on day 0, which the CBF was measured immediately after stroke, indicated that all the animals received an equal amount of ischemic insult. On day 2, the CBF of vehicle mice was 39.5 ± 6.2% in the peri‐infarct area, which was increased by EPC‐EX infusion (52.1 ± 7.3%, vs vehicle; *P* < 0.05). On day 14, the CBF was 53.6 ± 8.8% which was increased to 81.6 ± 8.2% by EPC‐EX infusion. EPC‐EXs^miR126^ was more effective in preserving CBF (73.8 ± 6.5% for day 2; 92.3 ± 4.4% for day 14, EPC‐EXs^miR126^ vs EPC‐EXs; *P* < 0.05).

As shown in Figure 4C, when compared to the contralateral, the density of MVD in the peri‐infarct area of nontreated mice (vehicle) was decreased by about 50% (*P* < 0.05). Infusion of EPC‐EXs was able to increase the MVD in the peri‐infarct area on both day 2 and day 14. Similarly, the infusion of EPC‐EXs^miR126^ further significantly induced an increase of the density MVD in the peri‐infarct area on both time points (*P* < 0.05 vs EPC‐EXs).

### 3.4 Infusion of EPC‐EXs^miR126^ is more effective than EPC‐EXs in improving neurologic function in diabetic stroke mice

**Figure 5:**
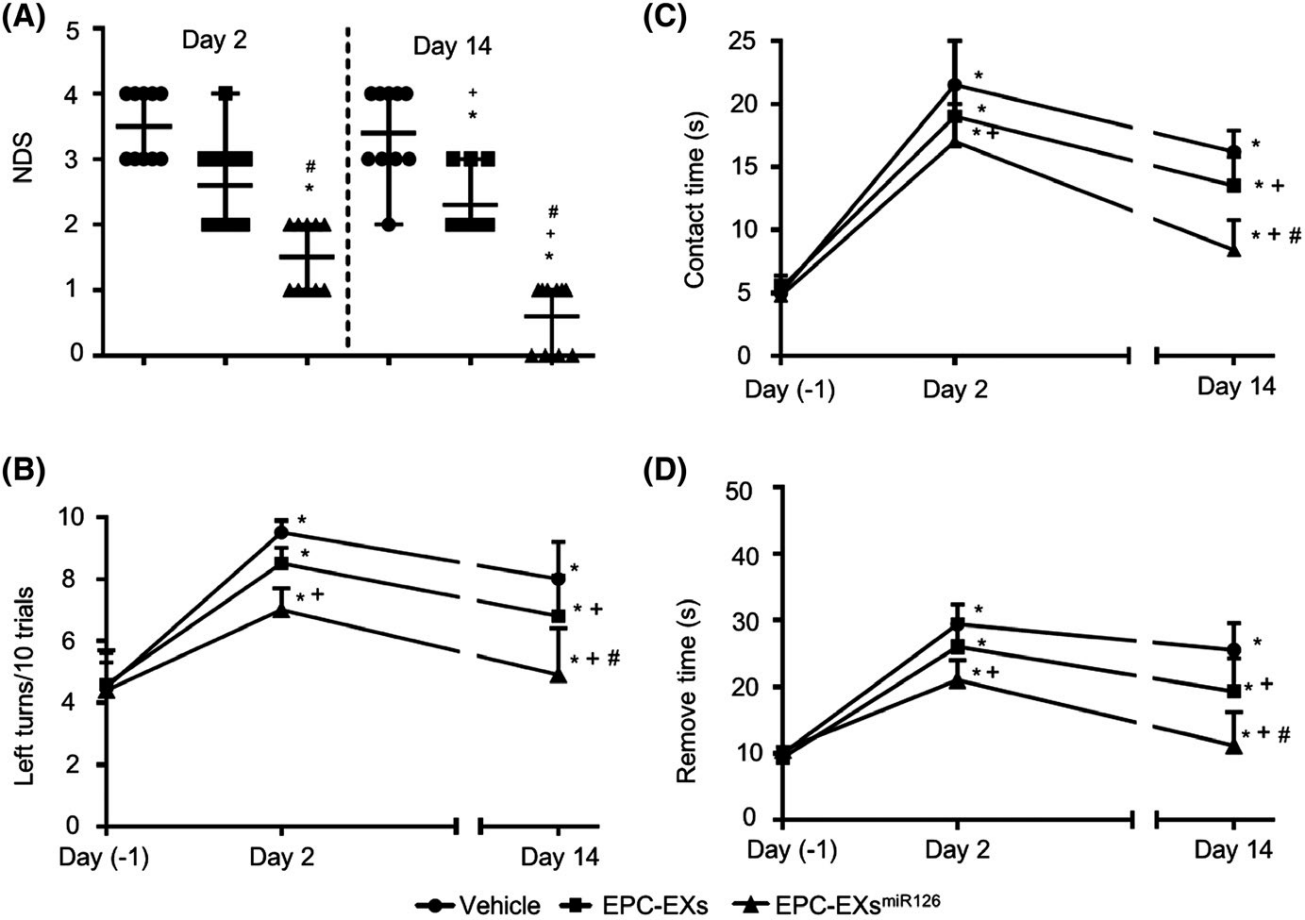
The effects of EPC‐EXs^miR126^ on NDS and sensorimotor functional recovery following ischemic stroke in diabetic mice. A: NDS in different treatment groups following MCAO‐induced ischemic stroke. The NDS was evaluated using the 5‐point scale method. *P < 0.05 vs vehicle; ^#^ *P* < 0.05 vs Day 2; ^+^ *P* < 0.05 vs EPC‐EXs; n = 10/group. B: Summarized data showing the number of left turns out of 10 turn trials in the different groups based on the corner test. C‐D: Summarized data showing the time‐to‐contact and time‐to‐remove of the tap in the adhesive removal test in different groups. *P < 0.05 vs day‐1; ^+^ *P* < 0.05 vs vehicle; ^#^ *P* < 0.05 vs EPC‐EXs; n = 11/group. Data are displayed as mean ± SD

As shown in Figure 5A, EPC‐EX infusion significantly improved the neurologic deficit score on both day 2 [3 (2‐4) and 3.5 (3‐4), *P* < 0.05] and day 14 [2 (2‐3) and 3.5 (3‐4); vs vehicle; *P* < 0.05], which were further improved by EPC‐EXs^miR126^ [2 (1‐2) for day 2 and 1 (0‐1) for day 14; vs EPC‐EXs; *P* < 0.05].

The sensorimotor function recovery was assessed on the day before MCAO surgery, 2 days and 14 days after the surgery. According to the results of the corner test (Figure 5B), we found that EPC‐EXs treatment did not significantly enhance the sensorimotor functional recovery on day 2. This is indicated by no overt change in the number of left turns out of every 10 turn trials as compared to that in the vehicle group (vs vehicle; *P* > 0.05). However, EPC‐EXs^miR126^ offered a beneficial effect on day 2 (vs vehicle; *P* < 0.05). On day 14, both EPC‐EXs and EPC‐EXs^miR126^ improved the sensorimotor function of the mice as revealed by decreased the number of lefts turns out of every 10 turn trials, with a better effect elicited by EPC‐EXs^miR126^ (*P* < 0.05).

Similarly, as shown in Figure 5C,D, on the affected (right) side, there was no significant difference in the time‐to‐contact and time‐to‐remove the adhesive tape between the mice in the vehicle and EPC‐EXs groups on day 2 (*P* > 0.05), but EPC‐EX^miR126^ treatment elicited a slight effect as compared to the vehicle group on day 2 (*P* < 0.05), while on day 14, the time‐to‐contact and time‐to‐remove the adhesive tape were significantly shortened in those mice treated by EPC‐EXs as compared to the vehicle group (*P* < 0.05). Furthermore, better effects were observed in those treated by EPC‐EX^miR126^ (*P* < 0.05). These data suggest that there was a better sensorimotor functional recovery with EPC‐EX^miR126^ treatment.

### 3.5 Infusion of EPC‐EXs^miR126^ is more effective than EPC‐EXs in promoting angiogenesis and neurogenesis in the peri‐infarct area in diabetic stroke mice

**Figure 6:**
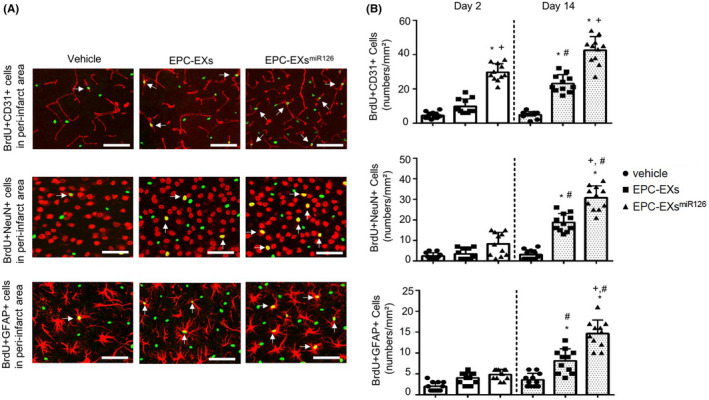
The effects of EPC‐EXs^miR126^ on angiogenesis and neurogenesis following ischemic stroke in diabetic mice. A: Representative images of angiogenesis (BrdU+/CD31 + cells) and neurogenesis (BrdU+/NeuN+ and BrdU+/GFAP + cells) on day 14. Arrows indicate the co‐localization of BrdU with endothelial cells, neurons, and astrocytes. Scale bars: 80 µm for BrdU+/CD31+; 40 µm for BrdU+/NeuN+ and BrdU+/GFAP+. B: Summarized data on angiogenesis and neurogenesis. *P < 0.05 vs vehicle; ^#^ *P* < 0.05 vs Day 2; ^+^ *P* < 0.05 vs EPC‐EXs; n = 11/group. Data are displayed as mean ± SD

Figure 6A shows representative micrographs of angiogenesis (BrdU + CD31 + cells) and neurogenesis (BrdU + NeuN + cells, BrdU + GFAP + cells) in the peri‐infarct area on day 14 after ischemic stroke. EPC‐EX infusion exhibited a tendency of promoting angiogenesis on day 2 (vs vehicle; *P* > 0.05) and significantly increased angiogenesis on day 14 (vs vehicle; *P* < 0.05). There was no significant difference between vehicle and EPC‐EX groups in neurogenesis on day 2, but on day 14 (vs vehicle; *P* < 0.05). Infusion of EPC‐EXs^miR126^ was more effective in promoting angiogenesis and neurogenesis (vs EPC‐EXs; *P* < 0.05, Figure 6B) on day 14.

### 3.6 Infusion of EPC‐EXs^miR126^ is more effective than EPC‐EXs in decreasing cell apoptosis in the brain of diabetic stroke mice

**Figure 7:**
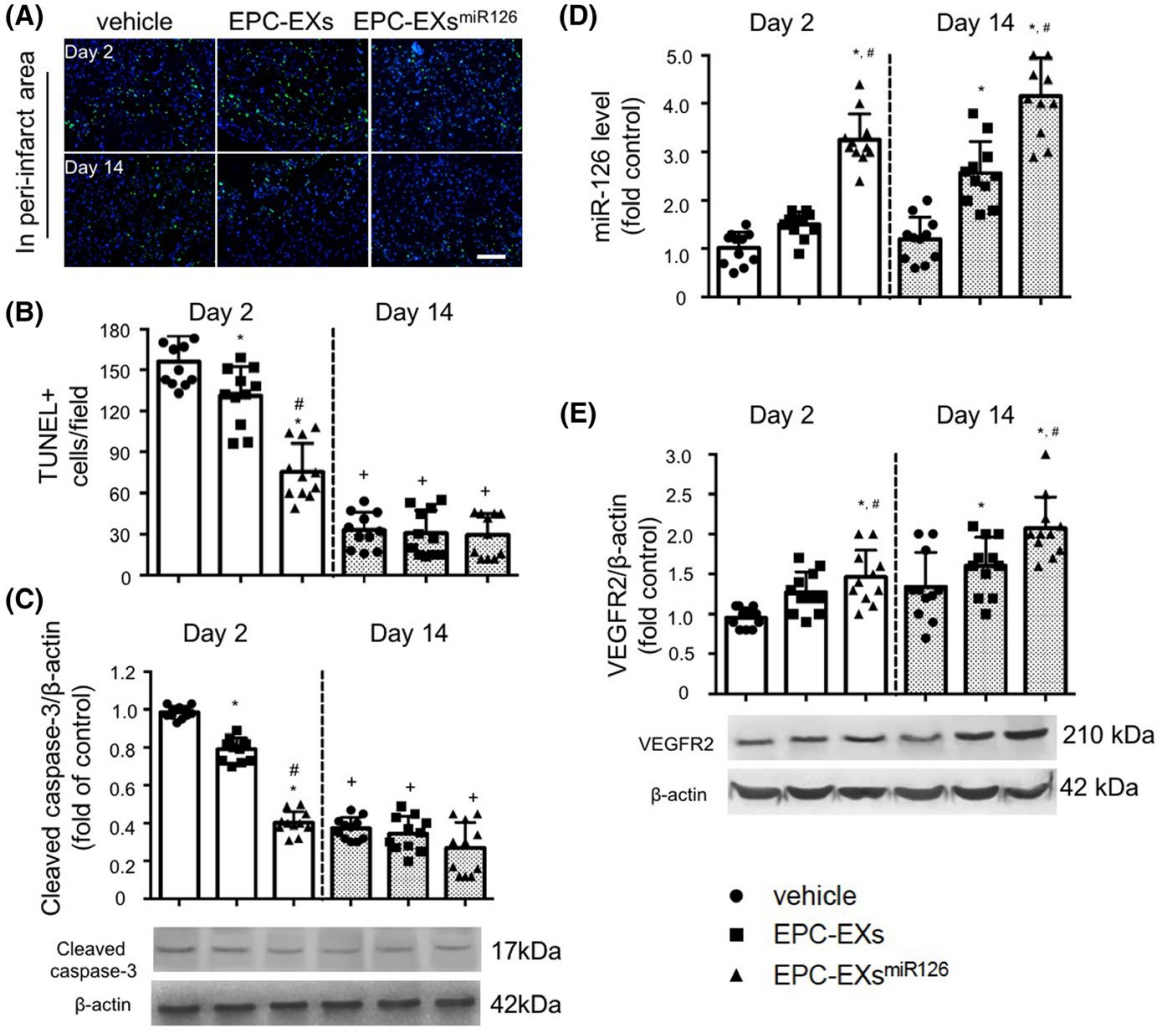
The effects of EPC‐EXs^miR126^ on cell apoptosis and the levels of miR‐126 and VEGFR2 in the brain following ischemic stroke in diabetic mice. A: Representative images of TUNEL + cells in different groups. Green: TUNEL staining; Blue: DAPI for cell nucleus. Scale bars: 100 µm. B: Summarized data on the numbers of TUNEL + cells. C: Western blot analysis of cleaved caspase‐3 expression in the brain on the stroke size after different treatments following ischemic stroke. D: Real‐time PCR analysis of miR‐126 expressions in the brain of the stroke side after different treatments. Small nuclear RNA U6 was used as an internal control. E: Western blot analysis of VEGFR2 expression in the brain of the stroke side after different treatments following ischemic stroke. β‐actin was used to normalize the protein loading. *P < 0.05 vs vehicle; ^#^ *P* < 0.05 vs EPC‐EXs; ^+^ *P* < 0.05 vs Day 2; n = 11/group. Data are displayed as mean ± SD

As shown in Figure 7A‐C, and Figure S1, numbers of TUNEL + cells in the brain were dramatically increased on day 2 after MCAO and could be detected until day 14. EPC‐EX infusion could slightly decrease the numbers of TUNEL + cells (by ~ 1.1‐fold, vs vehicle; *P* < 0.05), while EPC‐EXs^miR126^ significantly decreased the numbers of TUNEL + cells on day 2 (by ~ 2.1‐fold, vs EPC‐EXs; *P* < 0.05). However, either EPC‐EXs or EPC‐EXs^miR126^ did not have effects on cell death on day 14 (*P* > 0.05). To further distinguish the apoptosis from necrosis, the expression of cleaved caspase‐3 in the brain was measured. Similarly, the level of cleaved caspase‐3 was detected on day 2 and dramatically decreased on day 14 after MCAO (*P* < 0.05). EPC‐EXs^miR126^ was more effective than EPC‐EXs in decreasing the expression of cleaved caspase‐3 in the brain on day 2 (*P* < 0.05), but not on day 14 (*P* > 0.05) after MCAO. The data suggest that EPC‐EXs^miR126^ is more effective than EPC‐EXs in protecting the brain from acute ischemic injury by decreasing ischemia‐induced cell apoptosis through a caspase‐dependent pathway.

### 3.7 Infusion of EPC‐EXs^miR126^ is more effective than EPC‐EXs in increasing the level of miR‐126 and VEGFR2 in the brain of diabetic stroke mice

EPC‐EX infusion increased the level of miR‐126 on day 14 but not on day 2 (vs vehicle; *P* > 0.05). EPC‐EXs^miR126^ infusion could significantly increase the miR‐126 levels on both day 2 and day 14 (vs EPC‐EXs; *P* < 0.05). As revealed by western blot analysis, EPC‐EX infusion increased the level of VEGFR2 in the brain on day 14 (*P* < 0.05), but not on day 2 (vs vehicle; *P* > 0.05). Interestingly, EPC‐EXs^miR126^ infusion increased the VEGFR2 levels (vs vehicle; *P* < 0.05) on day 2 and prolonged the high level of VEGFR2 till day 14 after MCAO (vs EPC‐EXs, *P* < 0.05; Figure 7D,E). These data suggest that EPC‐EXs^miR126^ is more effective than EPC‐EXs in promoting angiogenesis and facilitating neurogenesis in the chronic phase by upregulating VEGFR2.

## 4 DISCUSSION

In diabetic patients, ischemic cerebral damage is exaggerated, and the outcome is poor. Our previous study showed that the infarct size of ischemic stroke was enlarged in diabetic mice which could be reduced by the administration of EPCs.^6^, ^20^ The mechanism associated with this reduction might be ascribed to EPC‐released growth factors and EXs. In the present study, we focused on diabetic mice to see whether EPC‐released EXs could be one of the mechanisms underlying the therapeutic effects of EPCs on diabetic mice. Our data showed that EPC‐EXs could protect the brain from acute injury and promote cerebral repair in db/db type‐2 diabetic mice and further explored the underlying mechanism. These data provide evidence to support the conception that EXs released by transplanted cells can mediate the benefits of cell‐based therapy.

The ischemic penumbra is the zone of tissue between the infarct core and normal brain, which suffers diminished blood flow but preserves cellular metabolism. The protection of the peri‐infarct area is critical for ischemic stroke therapy. Herein, we demonstrated that EPC‐EX infusion could enter the brain and rescue the cells in the peri‐infarct or penumbra area. The dose we used for EPC‐EXs is 1 × 10^11^ in 100 µL PBS which is comparable to 50 μg total EX protein determined by our published data.^21^ This dose is based on a previous study that showed beneficial effects of EPC‐released microvesicles on hindlimb ischemia in a murine model.^15^ Indeed, the intravenous administration of PKH26 labeled EPC‐EXs could cross the brain‐blood barrier and dominantly merged with ECs, astrocytes, and neurons in the penumbra area. There are studies have shown that EXs enriched with proinflammatory signal molecules such as miRs could be incorporated into microglial and thereby alter the inflammation status in the brain.^30^, ^31^ In the present study, we discovered for the first time that the injected EPC‐EXs could also be uptaken by microglia.

In a diabetic stroke, overloads anaerobic energy production results from hyperglycemia can cause stress on neurons and vascular endothelial cells, exacerbate reactive oxidative stress accumulation thereby leading to increased cell death upon ischemic injury.^32^ EPC‐derived microvesicles have been demonstrated to elicit antiapoptotic and antioxidative effects on endothelial cells and cardiomyocytes.^18^, ^22^ In the present study, we found that the total number of apoptotic cells in the penumbra area was decreased after EPC‐EXs injection. The EPC culture medium containing EXs has been shown to suppress neural apoptosis in a traumatic spinal cord injury rat model.^33^ We assumed the injected EPC‐EXs could protect neurons from ischemia‐injured apoptosis in our experimental model, although we did not verify the specific cell types undergoing apoptosis. These neurovascular protective effects were specific for the acute phase, as the apoptosis of brain cells and the level of caspase‐3 were decreased on day 2 after treatment. It has become widely accepted that extensive apoptosis is responsible for exaggerated damage and poor outcome in IS.^34^ In this study, we examined the infarct volume, MVD, and CBF in the penumbra area. The significant reduction in infarct volume and the preservation of MVD and CBF suggest a neurovascular protection effect on ischemic stroke in the acute phase.

To confirm the therapeutic effects of EPC‐EXs on ischemic stroke in the chronic recovery phase, we evaluated the angiogenesis and neurogenesis after treatment on day 14. Angiogenesis is a key procedure of tissue repair in the chronic phase of ischemic stroke. EPC‐released microvesicles are found to play a vital role in angiogenesis.^15^, ^35^ In the present study, newly generated ECs (CD31 + BrdU + cells) were measured for angiogenesis as we previously reported.^6^, ^7^ In the EPC‐EX infusion group, the level of newly generated ECs is increased in the penumbra area on day 14. Besides angiogenesis, neurogenesis is a potential target for treating ischemic stroke. Previous reports have shown that angiogenesis facilitates the neuroprotection and neurogenesis in the recovery phase of ischemic stroke.^36^, ^37^, ^38^ The blockade of angiogenesis delays the process of neurogenesis^38^ because the reformed blood vessels restore nutritive blood flow for neurons.^39^, ^40^ On this basis, we further investigated whether EPC‐EX infusion also promotes neurogenesis in ischemic stroke. As we expected, EPC‐EXs promoted neurogenesis on day 14. The underlying mechanisms of such effect could be ascribed to their carried molecules, which subsequently modulate their pathways.^15^, ^16^, ^35^ EXs contain cargoes like miRs, which can be delivered to recipient cells and modify the target cell functions.^8^, ^9^ A previous study showed that that EXs from mesenchymal stem cells mediated the miR‐133b transfer to astrocytes and neurons in stroke rats.^41^ Of note, our data showed that EPC‐EXs promoted astrocyte proliferation as revealed by a higher level of BrdU + GFAP + cells in the peri‐infarct area. The astrocyte response to ischemia has traditionally been viewed as detrimental as glial scar formation, while some studies have suggested that astrocytes also respond to ischemia with functions important for neuroprotection and repair, which supports our finding. For example, systemic infusion of bone marrow stromal cells following MCAO increased gliogenesis and decreased lesion size.^42^, ^43^ Taken together, we demonstrated that EPC‐EXs could provide therapeutic effects on ischemic stroke in diabetic mice by delivering their carried miR‐126.

It is known that miR‐126 governs vascular function and promotes angiogenic process.^44^ Meanwhile, the decrease of miR‐126 has been found in diabetes^45^ and is related to the impaired proangiogenic ability.^46^ Little is known at present about the therapeutic efficiency of EPC‐EXs with miR‐126 overexpression in diabetic ischemic stroke. It is supposed that the enrichment of miR‐126 in EPC‐EXs could enhance the therapeutic effects on ischemic stroke in diabetic mice. To answer this question, we transfected EPCs with miR‐126 mimics to obtain miR‐126 enriched EPC‐EXs. The level of miR‐126 in EPC‐EXs was confirmed to increase by ~ 6‐fold. Functional studies were conducted after the tail vein injection of EPC‐EXs into diabetic ischemic stroke mice. It is interesting to note that EPC‐EXs^miR126^ infusion significantly raised the miR‐126 level in the brain, suggesting the effective transferrin of miR‐126 to the brain by EPC‐EXs^miR126^. Meanwhile, our data showed that the EPC‐EXs level in the ipsilateral brain was raised. This could be explained by the chemoattraction effect induced by stromal cell‐derived factor 1α (SDF‐1α) and express CXC chemokine receptor type 4 (CXCR4). The enriched expressions of SDF‐1α/CXCR4 in EPC‐EXs^miR126^ (data not shown) could attract more EPCs into the infarct area and thereby releasing more EXs. More importantly, our outcome data revealed that EPC‐EXs^miR126^ transplantation was more effective than EPC‐EXs in decreasing infarct volume and preserving MVD and CBF in the acute phase. This is supported by a previous report showing that miR‐126 overexpression could decrease apoptosis and reactive oxygen species production in ECs.^47^ In the later repair phase, EPC‐EXs^miR126^ had better efficiency than EPC‐EXs in promoting angiogenesis and neurogenesis. Moreover, the neurological functions data showed that the EPC‐EXs^miR126^ had enhanced efficiency in decreasing NDS and improving the sensorimotor functions. Taken together, miR‐126 boosts the protective and therapeutic effects of EPC‐EXs on ischemic stroke in diabetic mice in both acute and chronic recovery phases.

Mechanistically, the beneficial effects could be from both EPC‐EXs and their carried miR‐126 by modulating different signaling pathways. Cantaluppi et al^16^ have demonstrated that EPC‐released microvesicles could protect ECs from hypoxia‐induced apoptosis by downregulating inflammatory and proapoptotic pathways. We previously demonstrated that EPC‐released microvesicles shuttle mRNAs and miRs were involved in cell viability, angiogenesis, and proliferation.^18^, ^22^ We recently found that EPC‐EXs were associated with the angiogenic pathways such as eNOS and Nox,^48^ as well as the mitochondrial and apoptotic pathways.^49^ We now identified that the neurovascular protection effects of EPC‐EXs and EPC‐EXs^miR126^ in acute ischemic stroke by decreasing apoptosis were from the modulation of the Caspase‐3 pathway. The role of miRs shuttled by EPC‐EXs in renal cell regeneration suggests that miRNAs delivered by EPC‐EXs contribute to their regenerative potential.^50^ Moreover, miR‐126 was recognized to have an important role in EPC‐EXs‐associated vascular and neural protective effects, as EXs derived from EPCs treated with miR‐126 inhibitors were less effective.^19^ As one of the miR‐126 downstream pathways, VEGFR2 is responsible for most downstream angiogenic effects of VEGF.^51^ An early study has shown that VEGF to VEGFR2 binding could activate downstream survival and migration pathways like PI3‐kinase/Akt and focal adhesion kinase.^52^ In a stroke, the expression of VEGFR‐2 in the vasculature was increased in the peri‐infarct area compared with the contralateral hemisphere.^53^ The upregulation of VEGFR‐2 contributes to the neuro‐vascularization in the penumbra,^54^, ^55^ which is in agreement with our data showing improved angiogenesis and neurogenesis in the peri‐infarct area of mice treated by EPC‐EXs. However, since VEGFR2 could be expressed on different types of cells such as vascular endothelial cells, neurons, and neuroblast,^56^ the types of brain cells which are primarily responsible for the increased VEGFR2 expression in our animal model requires further study. We have previously discovered that miR‐126 regulated the angiogenic process and EC/EPC function by modulating VEGFR2‐related signal transduction.^57^, ^58^ In the present study, we revealed that the potential mechanisms for the beneficial effects elicited by EPC‐EXs and EPC‐EXs^miR126^ on ischemic stroke in the recovery phase were probably through the VEGFR2 pathway. Our finding is also supported by another study showing that VEGF‐receptors start to be upregulated as early as 2‐4 hours after the onset of stroke and to last for at least 28 days.^54^

In summary, enrichment of miR‐126 in EPC‐EXs provides a positive strategy to enhance the therapeutic effects of EPC‐EXs on diabetic ischemic stroke by protecting the brain from acute injury and promoting neurological functional recovery via accelerating angiogenesis and neurogenesis. This may lead to a novel cell‐free therapeutic approach for diabetic stroke.

## CONFLICT OF INTEREST

The authors declare no conflict of interest.

## Supporting information

Fig S1

## Data Availability

The data that support the findings of this study are available from the corresponding author upon reasonable request.
