# Supplementary figures and images for: Exosomes from miRNA‐126‐modified endothelial progenitor cells alleviate brain injury and promote functional recovery after stroke
Source: CNS Neurosci Ther. 2020 Oct 3;26(12):1255–65. doi: 10.1111/cns.13455 (PMC7702230; doi:10.1111/cns.13455)

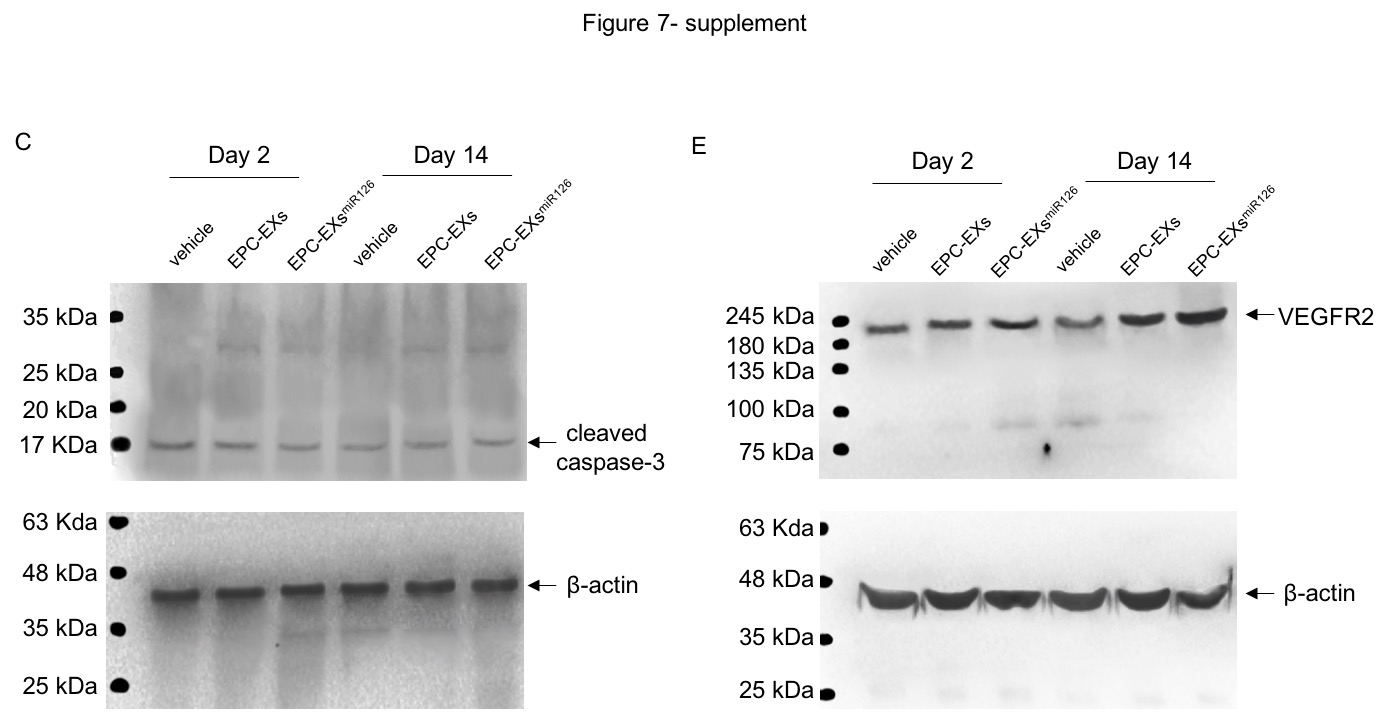

Supplement: Supplementary file 1 — Fig S1 [file CNS-26-1255-s001.jpg]
